# Electrophysiological responses to images ranging in motivational salience: Attentional abnormalities associated with schizophrenia-spectrum disorder risk

**DOI:** 10.1038/s41598-020-61504-2

**Published:** 2020-03-12

**Authors:** Elizabeth A. Martin, Lilian Yanqing Li, Mayan K. Castro

**Affiliations:** Department of Psychological Science, University of California, Irvine, Irvine, California USA

**Keywords:** Psychology, Human behaviour

## Abstract

Individuals at risk for schizophrenia-spectrum disorders display abnormalities related to motivational salience, or the ability of stimuli to elicit attention due to associations with rewards or punishments. However, the nature of these abnormalities is unclear because most focus on responses to stimuli from broad “pleasant” and “unpleasant” categories and ignore the variation of motivational salience within these categories. In two groups at risk for schizophrenia-spectrum disorders—a Social Anhedonia group and a Psychotic-like Experiences group—and a control group, the current study examined event-related potential components sensitive to motivational salience—the Early Posterior Negativity (EPN), reflecting earlier selective attention, and the Late Positive Potential (LPP), reflecting sustained attention. Compared to controls, the Social Anhedonia group showed *smaller* increases in the EPN in response to erotica and smaller increases in the LPP as the motivational salience of pleasant images increased (exciting<affiliative<erotica). In contrast, the Psychotic-like Experiences group had *larger* increases in LPP amplitudes as the motivational salience of pleasant images increased. Also, both at-risk groups showed larger increases in the LPP to threatening images but smaller increases to mutilation images. These findings suggest that examining abnormalities beyond those associated with broad categories may be a way to identify mechanisms of dysfunction.

## Introduction

Affect and motivation are intrinsically linked^1^. For example, motivational salience, or the ability to elicit attention due to associations with rewards and punishments, is an intrinsic quality of affective stimuli^2^. Motivational salience plays a critical role in the brain’s initial dopamine response, and subsequently, influences behaviors and judgments. As evidence of the link between affect and motivational salience, individuals with emotional deficits, such as people with schizophrenia and schizophrenia-spectrum personality disorders, typically also display abnormalities related to motivational salience^3,4^. These affect-motivation salience abnormalities are associated with a host of poor outcomes related to behaviors and judgments of these groups, such as impaired social functioning and work performance^5,6^.

Similarly, people at risk for schizophrenia-spectrum disorders (i.e., “at-risk groups”), such as people with extremely high levels of social anhedonia^7,8^, or people who report increased frequency of psychotic-like experiences (e.g., increased frequency of perceptual aberrations and magical ideation)^9^, show abnormalities related to the motivational salience of affective stimuli. For example, social anhedonia, which is characterized by diminished self-reported experience of positive emotion e.g.^10–12^, is associated with decreased self-reported attention to positive emotions^13,14^, as well as decreased influence of emotion on behavioral judgments^12,15^. This suggests that *pleasant stimuli might have a reduced motivational salience for individuals with social anhedonia*. In contrast, there is some evidence that individuals with psychotic-like experiences show greater attention to positive stimuli. For example, Martin and colleagues^16^ found larger electrophysiological reactivity, indexed by the Late Positive Potential (LPP), in response to pleasant images for a Psychotic-like Experiences group compared to a Social Anhedonia and Control group. This suggests that *pleasant stimuli might have an enhanced motivational salience for individuals with psychotic-like experiences*.

At the same time, there is evidence of increased salience of unpleasant stimuli in at-risk groups. For example, both groups are associated with increased self-reported attention^12^ as well as increased physiological reactivity (e.g., heart rate) and LPP amplitudes, in response to negative stimuli compared to control groups^16,17^. Also, there is evidence of increased goal-directed behavior if importance is attributed to any event or object for individuals with psychotic-like experiences^18,19^. Taken together, this suggests an *enhanced motivational salience for unpleasant stimuli for individuals at risk for a schizophrenia-spectrum disorder in general*.

However, most previous investigations of emotional processing in at-risk populations have focused on responses to broad “pleasant” and “unpleasant” categories only e.g.^12,20–23^. This approach assumes that stimuli within a broad category have the same motivational salience, which is not the case^1,24^, and does not allow for an understanding of how differences in reward and punishment values among different pleasant and unpleasant stimuli might influence responses. Because understanding the nature of abnormalities related to motivational salience in at-risk individuals could help treat functional disability in people with a schizophrenia-spectrum disorder and aid in prevention efforts for those at risk, the current study sought to clarify whether electrophysiological responses varied as the motivational salience of stimuli increased within a broad category in at-risk groups compared to a control group.

Within broad affect categories, there are subcategories of images with a wide range of content that varies in levels of motivational salience^1,24^. That is, as subcategories become increasingly related to survival (e.g., procreation, death), they elicit increased attention due to associations with rewards or punishments. Subsequently, these increases are associated with distinct physiological outcomes but not necessarily significant differences in self-reported affect ratings. For example, within the pleasant category, both erotic images (e.g., people kissing) and exciting images (e.g., a person windsailing) are rated as “pleasant”. However, electrophysiological responses reveal an important difference between the two subcategories: responses to erotic images are larger than responses to exciting images in healthy people^25^. Similarly, within the unpleasant category, both mutilation images (e.g., severed body parts) and disgusting images (e.g., a dirty toilet) are rated as “unpleasant”, but electrophysiological responses show differential responding between the two subcategories: responses to mutilation images are larger than responses to disgusting images in healthy people^25^. This suggests that as the motivational salience of subcategories of images within a broad category increases, the magnitude of elicited event-related potential (ERP) responses also increases. Thus, studying responses to images within these specific subcategories could lead to a more refined understanding of abnormalities in at-risk groups. Specifically, if at-risk groups show abnormalities related to motivational significance, we would expect that as the motivational significance of images increases, abnormalities would become more apparent.

To objectively assess responses of at-risk populations to images with varying motivational salience, we examined two ERP components—the Early Posterior Negativity (EPN) and the LPP. For healthy individuals, both the EPN and the LPP are larger in amplitude in response to valenced stimuli compared to neutral stimuli e.g.^26,27^ and have also been shown to be sensitive to the motivational salience of stimuli within a broad valence category^25,28^. Larger amplitudes of the EPN and LPP in response to valenced compared to neutral stimuli have been interpreted as a reflection of increased attention to motivationally relevant stimuli e.g.^26,29–31^. However, as discussed below, the EPN and LPP are also thought to reflect different stages of attentional processing. Thus, given the temporal precision of the ERP methodology^32^, we can identify not only whether abnormalities related to increasing motivational significance exist in at-risk groups, but also at what stage of attentional processing they occur.

The EPN, which is a temporo-occipital, negative deflection that peaks at approximately 230 ms post-stimulus onset^32^, has been suggested to reflect the enlistment of early, selective attentional processes^30^. In contrast, the LPP, which is a centro-parietal, positive-going waveform starting at around 300 ms post-stimulus onset, is thought to reflect the sustained attention to, and continued processing of, motivationally relevant stimuli e.g.^27^. Given evidence that the LPP can extend for 1,000 ms or beyond stimulus-onset and that there may be differences in responding over this period^26^, researchers often examine early and later windows of the LPP. If at-risk groups have a deficit in early attentional processes, we would expect them to have smaller EPN responses (i.e., less negative/more positive amplitudes) compared to a control group. At the same time, if at-risk groups have decreased sustained processing, we would expect them to have smaller LPP responses compared to the control group.

In the current study, a Social Anhedonia group, a Psychotic-like Experiences group, and a control group passively viewed pleasant, unpleasant, and neutral images while electrophysiological activity was recorded. Within each broad emotion category, they viewed three subcategories of images that varied in motivational salience^29,33,34^. We tested whether there were group differences in changes in EPN or LPP amplitudes as a function increasing motivational salience of images. Based on previous evidence^25,28^, we predicted that the control participants would show larger ERP responses as the motivational salience of pleasant and unpleasant stimuli increased. Based on prior self-report, behavioral, and electrophysiological investigations of attention to pleasant emotion^16,21,35^, we predicted that the Social Anhedonia group would show smaller increases in ERP amplitudes as the motivational salience of pleasant stimuli increased compared to the other groups. In contrast, given evidence of increased reactivity to pleasant stimuli^16,17^, we predicted that the Psychotic-like Experiences group would show larger increases in ERP amplitudes as the motivational salience of pleasant stimuli increased compared to the other groups. At the same time, given evidence of increased attention and reactivity to unpleasant stimuli in at-risk groups e.g.^12,16^, we predicted that the at-risk groups would display larger increases in ERP amplitudes as the motivational salience of unpleasant stimuli increased. Also, based on some evidence that even neutral images can vary in levels of motivational salience^25^, we tested whether the at-risk groups exhibited differences in ERP amplitudes as the motivational salience of these images increased. Given its exploratory nature, we did not have a hypothesis regarding whether or how the groups’ ERP responses might differ in response to neutral images with increasing motivational salience. Finally, given previous evidence that at-risk groups display abnormalities related to more sustained attention^16^, we predicted that there would be LPP amplitude differences between the at-risk and Control groups in the current study. We further explore whether there were earlier selective attention abnormalities in at-risk groups, evidenced by deviant EPN amplitudes.

## Results

### Decreased early selective attention to pleasant images with high motivational salience in social anhedonia

**Figure 1:**
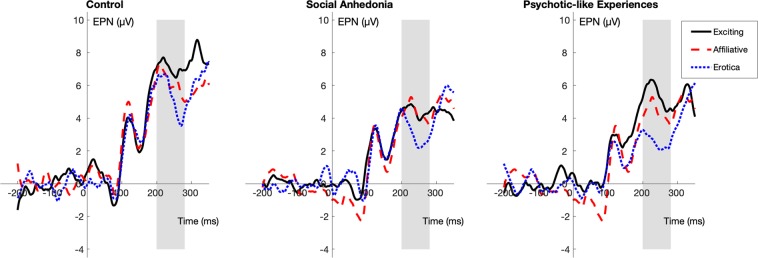
Stimulus-locked ERPs averaged at POz, Oz, O1, and O2 (EPN) for each of the pleasant subcategories (exciting, affiliative, erotic) by group. The gray bar indicates the portion of the waveform used in analysis.

As can be seen in Fig. 1 and Table 1, with exciting images as the reference, the Social Anhedonia group showed significantly smaller increases in EPN amplitudes (i.e., less negative) compared to the Control group in response to erotic images (β_partial_ = 0.33, SE = 0.16, *t*_679_ = 2.08, *p* = 0.03). This suggests that the Social Anhedonia group showed decreased early selective attention to images with high motivational salience. No other group difference in early selective attention to pleasant or unpleasant images reached statistical significance. Please see the Supplementary Materials (Supplementary Table 1) for full multi-level model results.

**Table 1:** Mean Amplitudes (μV) of EPN (200–280 ms), Early LPP (400–1000 ms), and Late LPP (1000–1500 ms) in Response to Images by Group.

	Control			Social Anhedonia			Psychotic-like Experiences		
	EPN	Early LPP	Late LPP	EPN	Early LPP	Late LPP	EPN	Early LPP	Late LPP
	(*n* = 22)	(*n* = 19)	(*n* = 15)	(*n* = 19)	(*n* = 18)	(*n* = 14)	(*n* = 25)	(*n* = 23)	(*n* = 9)
Pleasant Images									
Exciting	7.74 (7.03)	1.08 (6.53)	−1.16 (3.53)	4.42 (4.75)	0.35 (2.76)	−0.09 (2.40)	5.46 (5.19)	0.26 (4.30)	−0.17 (2.49)
Affiliative	6.56 (4.74)	2.18 (6.29)	0.21 (4.51)	4.39 (4.83)	2.00 (3.63)	0.03 (2.76)	4.30 (4.66)	2.97 (4.55)	0.30 (1.54)
Erotic	4.00 (7.55)	3.07 (7.08)	0.04 (5.65)	3.04 (3.92)	3.11 (4.22)	−0.56 (4.47)	2.51 (4.70)	4.79 (4.88)	0.85 (2.30)
Unpleasant Images									
Disgusting	5.95 (4.90)	2.34 (4.81)	0.23 (4.48)	3.05 (5.08)	0.70 (4.90)	0.28 (2.80)	5.82 (5.81)	2.46 (2.58)	1.63 (2.60)
Threatening	5.47 (6.98)	1.61 (6.62)	−0.35 (4.34)	3.87 (4.30)	4.43 (5.50)	1.16 (4.65)	5.29 (5.17)	3.67 (4.91)	0.40 (4.15)
Mutilation	5.70 (4.10)	5.25 (4.99)	3.34 (4.24)	3.06 (5.50)	2.87 (4.27)	0.44 (4.47)	4.72 (5.01)	4.62 (5.04)	2.34 (3.00)
Neutral Images									
Objects	5.18 (7.48)	−1.55 (3.89)	−1.45 (2.73)	4.84 (5.36)	−0.70 (2.83)	−0.31 (2.26)	4.70 (4.95)	−0.08 (3.85)	0.31 (3.04)
Scenes without people	6.92 (6.99)	−0.35 (5.80)	−0.98 (4.49)	4.67 (5.07)	0.06 (3.76)	−0.52 (2.53)	5.09 (5.74)	−0.72 (3.08)	−1.45 (3.94)
Scenes with people	5.59 (6.80)	0.64 (7.08)	−0.18 (7.08)	4.14 (5.24)	1.21 (7.19)	−0.01 (4.85)	3.70 (4.35)	0.85 (2.41)	0.67 (1.92)

### Divergent abnormalities related to sustained attention to pleasant images in the at-risk groups

**Figure 2:**
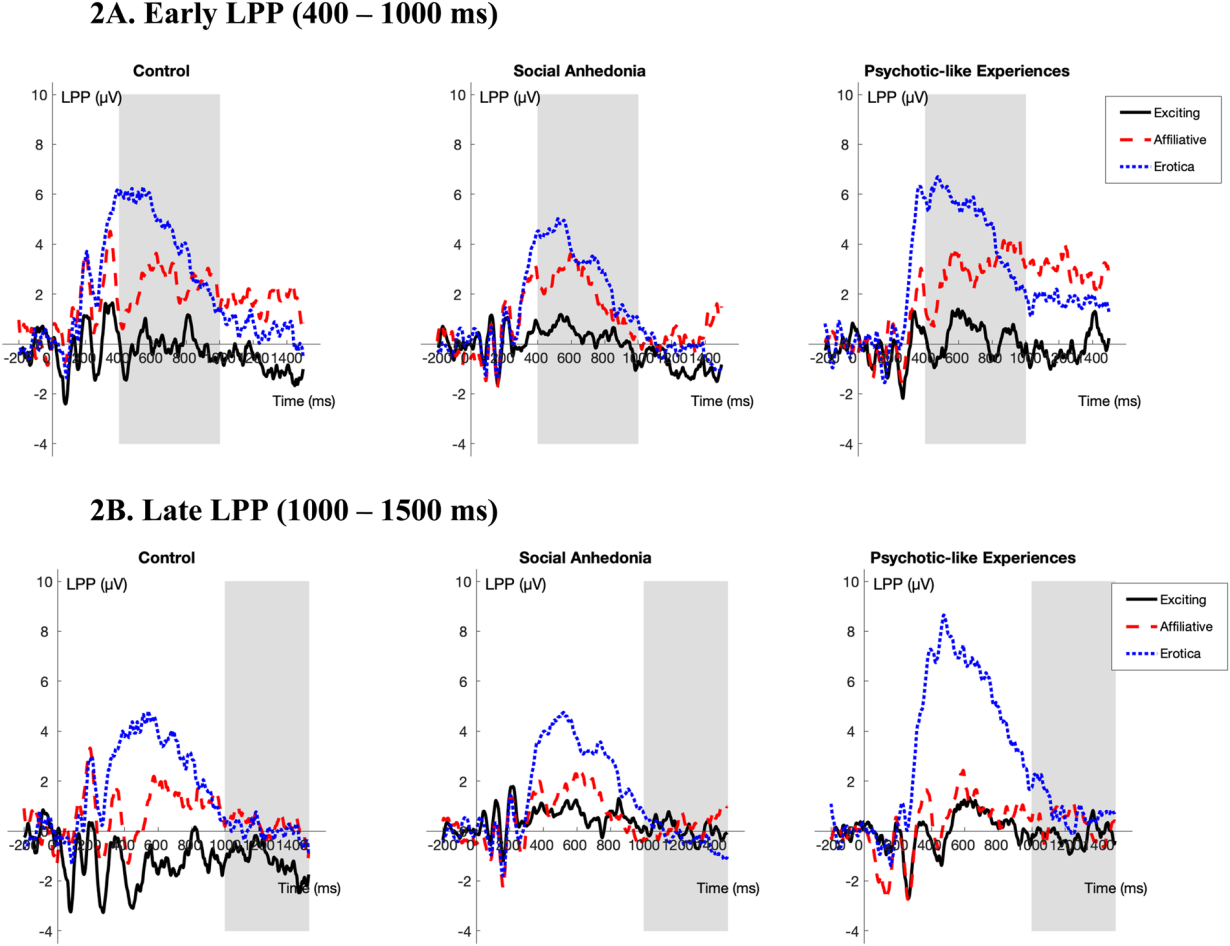
Stimulus-locked ERPs averaged at Pz, P3, P4, CPz, CP1, and CP2 (LPP) for each of the pleasant subcategories (exciting, affiliative, erotic) by group. The gray bar indicates the portion of the waveform used in analysis. (Note: Based on the dependability analyses, more participants were excluded from the Late LPP analyses due to an insufficient number of trials retained. Thus, there are separate figures for the Early and the Late LPP.).

As can be seen in Fig. 2, for the early LPP using exciting images as the reference, the Psychotic-like Experiences group showed larger increases in amplitudes compared to both the Control group (β_partial_ = 0.38, SE = 0.14, *t*_686_ = 2.73, *p* < 0.01) and Social Anhedonia group (β_partial_ = 0.32, SE = 0.14, *t*_688_ = 2.36, *p* = 0.02) in response to erotic images.

In contrast, as can been seen in Fig. 2 for the late LPP using exciting images as the reference, the Social Anhedonia group showed smaller increases in amplitudes compared to the Control group in response to affiliative (β_partial_ = −0.33, SE = 0.15, *t*_640_ = −2.25, *p* = 0.02) and erotic images (β_partial_ = −0.44, SE = 0.15, *t*_640_ = −2.99, *p* < 0.01). In addition, the Social Anhedonia group showed smaller increases in amplitudes compared to the Psychotic-like Experiences group in response to erotic images (β_partial_ = −0.41, SE = 0.16, *t*_640_ = −2.53, *p* = 0.01). No other group comparison of sustained attention to pleasant images was statistically significant. Taken together, these findings suggest that there are divergent abnormalities related to sustained attention to pleasant images in at-risk groups (i.e., compared to Control group, increased sustained attention to erotic images in the Psychotic-like Experiences group and decreased sustained attention to affiliative and erotic images in the Social Anhedonia group). Please see the Supplementary Materials (Supplementary Table 2 and 3) for full multi-level model results.

### Similar abnormalities related to sustained attention to unpleasant images in the at-risk groups

**Figure 3:**
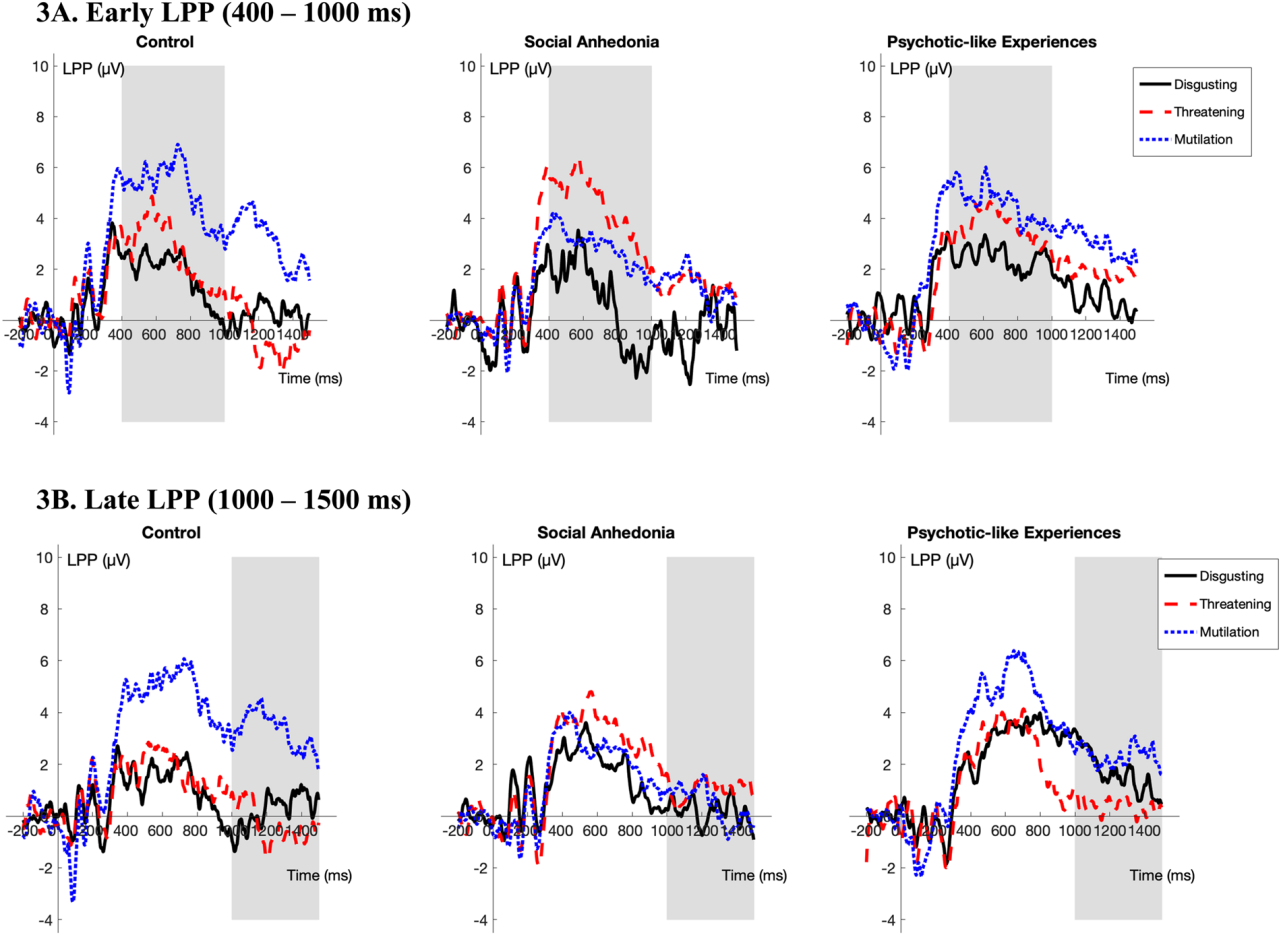
Stimulus-locked ERPs averaged at Pz, P3, P4, CPz, CP1, and CP2 (LPP) for each of the unpleasant subcategories (disgusting, threatening, mutilation) by group. The gray bar indicates the portion of the waveform used in analysis. (Note: Based on the dependability analyses, more participants were excluded from the Late LPP analyses due to an insufficient number of trials retained. Thus, there are separate figures for the Early and the Late LPP.).

As can be seen in Fig. 3, for the early LPP using disgusting images as the reference, both at-risk groups showed larger increases in amplitudes compared to the control group in response to threatening images (vs. Social Anhedonia group: β_partial_ = 0.70, SE = 0.14, *t*_997_ = 5.08, *p* < 0.001; vs. Psychotic-like Experiences group: β_partial_ = 0.36, SE = 0.13, *t*_993_ = 2.79, *p* < 0.01). Also, the Social Anhedonia group exhibited larger increases compared to the Psychotic-like Experiences group in response to threatening images (β_partial_ = 0.34, SE = 0.13, *t*_997_ = 2.59, *p* < 0.01).

Similar to results for the early LPP, as can be seen in Fig. 3 using disgusting images as the reference, the Social Anhedonia group showed larger late LPP amplitudes increases compared both other groups in response to the threatening images (vs. Control group: β_partial_ = 0.35, SE = 0.15, *t*_657_ = 2.26, *p* = 0.02; vs. Psychotic-like Experiences group: β_partial_ = 0.46, SE = 0.17, *t*_657_ = 2.71, *p* < 0.01). However, in contrast to the non-significant differences in early LPP amplitudes in response to mutilation images, both at-risk groups showed smaller late LPP amplitudes increases compared to the control group in response to mutilation images (vs. Social Anhedonia group: β_partial_ = −0.70, SE = 0.15, *t*_657_ = −4.57, *p* < 0.001; vs. Psychotic-like Experiences group: β_partial_ = −0.54, SE = 0.17, *t*_657_ = −3.21, *p* < 0.01). No other group comparison of sustained attention to unpleasant images was statistically significant. Taken together, these findings suggest that there are similar abnormalities in the at-risk groups related to sustained attention to unpleasant images (i.e., increased sustained attention to threatening images and decreased sustained attention to mutilation images). Please see the Supplementary Materials (Supplementary Table 2 and 3) for full multi-level model results.

### Largely similar responses to neutral images across groups

**Figure 4:**
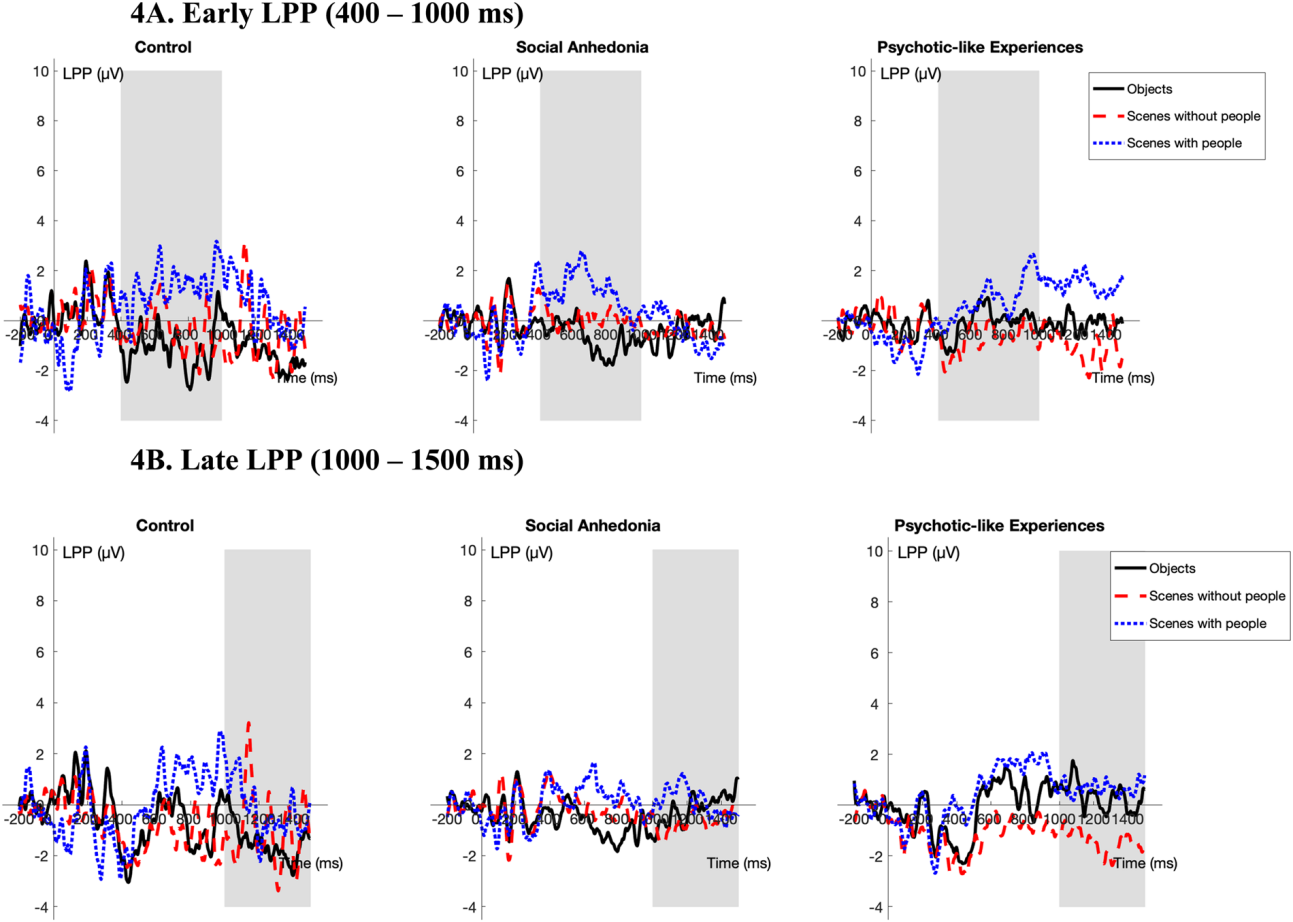
Stimulus-locked ERPs averaged at Pz, P3, P4, CPz, CP1, and CP2 (LPP) for each of the neutral subcategories (objects, scenes without people, scenes with people) by group. The gray bar indicates the portion of the waveform used in analysis. (Note: Based on the dependability analyses, more participants were excluded from the Late LPP analyses due to an insufficient number of trials retained. Thus, there are separate figures for the Early and the Late LPP.).

With images of objects as the reference, the Psychotic-like Experiences group exhibited smaller increases in late LPP amplitudes compared to the Control group (β_partial_ = −0.55, SE = 0.18, *t*_657_ = −2.98, *p* < 0.01) in response to scenes without people. As can be seen in Fig. 4, this suggests that responses to neutral images were largely consistent across groups. Please see the Supplementary Materials (Supplementary Table 1–3) for full multi-level model results.

### Image ratings

Please see the Supplementary Materials for full image rating results. Briefly, as can be seen in Table 2, there were no group differences in valence ratings for subcategories of pleasant, unpleasant, or neutral images, except for ratings for scenes without people: compared to the Control group, both the Social Anhedonia group (β_partial_ = −0.93, *t*_122_ = −2.41, *p* = 0.02) and the Psychotic-like Experiences group (β_partial_ = −0.67, *t*_121_ = −1.82, *p* = 0.07) gave lower ratings (i.e., more positive). Also, there were no group differences in arousal ratings for subcategories of pleasant or neutral images. For unpleasant images, The Social Anhedonia group gave lower arousal ratings for disgusting images relative to both the Control group (β_partial_ = −0.68, *t*_117_ = −2.29, *p* = 0.02) and the Psychotic-like Experiences group (β_partial_ = −0.61, *t*_115_ = −2.09, *p* = 0.04). The Psychotic-like Experiences group gave trend-level greater arousal ratings for threatening images compared to the Control group (β_partial_ = 0.47, *t*_128_ = 1.75, *p* = 0.08).

**Table 2:** Means (Standard Deviations) of image ratings by group.

	Control (*n* = 21)		Social Anhedonia (*n* = 21)		Psychotic-like Experiences (*n* = 25)	
	Valence	Arousal	Valence	Arousal	Valence	Arousal
Pleasant Images	3.07 (1.04)	5.24 (1.70)	3.57 (1.24)	4.78 (2.12)	3.20 (1.22)	5.36 (1.79)
Exciting	3.17 (1.50)	4.65 (1.67)	3.60 (1.50)	3.73 (1.94)	3.03 (1.15)	4.60 (1.96)
Affiliative	2.10 (1.26)	5.13 (2.49)	2.79 (1.79)	4.63 (2.82)	2.12 (0.97)	5.27 (2.24)
Erotic	4.12 (1.47)	5.76 (2.07)	4.71 (1.61)	5.81 (2.56)	4.02 (2.20)	6.41 (2.25)
Unpleasant Images	7.62 (0.95)	6.96 (1.34)	7.57 (1.24)	6.06 (2.08)	7.92 (0.67)	7.21 (1.38)
Disgusting	7.61 (1.25)	7.25 (1.32)	7.67 (1.26)	5.94 (2.44)	7.87 (0.92)	7.07 (1.56)
Threatening	7.25 (1.04)	6.19 (1.84)	7.24 (1.49)	5.37 (2.38)	7.64 (0.90)	6.95 (1.68)
Mutilation	7.97 (1.28)	7.57 (1.66)	7.94 (1.32)	7.01 (2.13)	8.17 (0.85)	7.50 (1.97)
Neutral Images	4.48 (0.92)	2.30 (0.82)	4.52 (0.91)	2.33 (1.20)	4.64 (0.56)	2.36 (1.08)
Objects	4.35 (1.30)	1.67 (0.66)	4.78 (0.58)	1.78 (1.14)	4.68 (0.69)	2.02 (1.26)
Scenes without people	4.83 (0.94)	2.64 (1.39)	4.21 (1.47)	2.21 (1.15)	4.45 (0.86)	2.45 (1.52)
Scenes with people	4.36 (1.29)	2.81 (1.53)	4.62 (1.06)	2.97 (2.10)	4.76 (0.83)	2.72 (1.27)

### Group differences in ERP amplitudes are not accounted for by arousal ratings of the images

In order to test whether any of the ERP results were due to differences in group arousal ratings to the images, we re-ran all of the models described above with the addition of relevant self-report arousal ratings as covariates (e.g., for the test of early LPP amplitudes in response to images within the broad pleasant category, we added self-report arousal ratings to the three subcategories of pleasant images). All of the patterns of results remained identical, including the size of the partially standardized estimates (e.g., for the early LPP using exciting images as the reference, the Psychotic-like Experiences group showed larger increases in amplitudes compared the Control group without arousal ratings in the model: β_partial_ = 0.38, SE = 0.14, *t*_686_ = 2.73, *p* < 0.01; with arousal ratings in the model: β_partial_ = 0.37, SE = 0.14, *t*_686_ = 2.732, *p* < 0.01). These results suggest that group differences in ERP amplitudes as the motivational salience of images increases are not due to differences in arousal ratings of the images.

## Discussion

The current study examined electrophysiological responses to subcategories of images that ranged in motivational salience in individuals at risk for developing schizophrenia-spectrum disorders and a control group. While the results for the control group were very consistent to the findings of Weinberg and Hajcak^25^, we found that the at-risk groups showed an abnormal, differential pattern of electrophysiological responses. Overall, these findings suggest that examining abnormalities in emotional functioning beyond those associated with the broad “pleasant” and “unpleasant” categories may be an important way to identify specific mechanisms of dysfunction in schizophrenia risk.

Consistent with our hypotheses, we found that the Social Anhedonia group showed decreased sustained attention to pleasant stimuli, indicated by smaller LPPs, as the motivational salience of the stimuli increased. This is consistent with findings from prior self-report, behavioral, and electrophysiological investigations of reduced attention to positive affect associated with anhedonia in both individuals with schizophrenia and those at risk e.g.^16,21,35^. This finding is also consistent with previous reports of negative associations between social anhedonia and sustained attention to pleasant stimuli, including elaborative processing^36^ or savoring of positive emotions^37^.

We also found that the Social Anhedonia group showed significantly smaller increases in EPN amplitudes (i.e., less negative) compared to the Control group in response to erotic images, evidence of reduced early selective attention to pleasant stimuli with high motivational salience. This suggests that attentional abnormalities related to pleasant affect stimuli are evident even earlier in the time course of processing than previously reported e.g.^16^. Taken together, these findings suggest that if individuals have chronically reduced attention to more motivationally salient pleasant stimuli, they may be less likely to engage in future, potentially pleasant events. Thus, attenuated attention to motivationally salient pleasant stimuli could be an intervention target for anhedonia.

One potential intervention strategy would be to use a dot probe task to ameliorate these attention deficits^38^. In the classic dot probe task, participants are instructed to fixate on the center of the computer screen and, after a short period of time, an affective stimulus and a neutral stimulus appear, one on each side of the screen. Then, the two images disappear, and one is replaced with a dot. Participants are instructed to indicate with the location of the dot with a button press as quickly as they can. Reaction times are interpreted as an indicator of where people’s attention is focused (e.g., if participants are faster at indicating the location of dots that replaced affective images, then their attention was largely focused on the affective stimuli over the neutral stimuli). With attentional retraining using this task, the dot always appears in the location of the stimuli that the trainers want participants to have a “bias” (e.g., in the location of the neutral stimuli vs. threatening stimuli in the case of retraining in anxiety disorders). Although the robustness of effects may vary with task nuances^39^, attentional biases toward or away from affective information can be modified in a single session^40^, with evidence that attention bias modification can have important real-world effects^41^. Hence, future research could use retraining on the dot probe task to attempt to increase attention to motivationally salient pleasant stimuli as a way to treat anhedonia.

In contrast to the findings related to social anhedonia and the motivational salience of pleasant stimuli, we found that the Psychotic-like Experiences group showed increased sustained attention to pleasant stimuli with high motivational salience, indicated by larger LPPs, compared to the other groups. This finding is consistent with previous evidence of increased physiological reactivity to pleasant stimuli in individuals with psychotic-like symptoms^16,17^. Relatedly, the Psychotic-like Experiences group in the current study was characterized by extremely high Perceptual Aberration scale^42^ and/or Magical Ideation scale^43^ scores. In addition to predicting future psychotic disorders, scores on these scales have also been predictive of future onset of bipolar disorders^9^ and disorders characterized by reward hypersensitivity^44^. The characterization of this group as having reward hypersensitivity is consistent with our results. Thus, our finding of increased sustained attention to pleasant stimuli for the Psychotic-like Experiences group is consistent with the broader literature. Overall, these findings indicate individuals with social anhedonia and individuals with psychotic-like experiences have unique emotional abnormalities related to ERP responses to pleasant images with high motivational salience.

As hypothesized, the at-risk groups showed larger increases in sustained attention in response to unpleasant images with moderate motivational salience (i.e., threatening images). This is consistent with previous literature suggesting Social Anhedonia and Psychotic-like Experiences groups show greater reactivity to negative stimuli^16,17^ and increased attention to negative emotion^12^. In addition, an increased electrophysiological response to threatening images specifically is consistent with the known positive association between symptoms of these groups and paranoia^45,46^. That is, these at-risk groups report high levels of paranoia, which is associated with increased vigilance and attention to threat-related stimuli. Thus, it is possible that threatening images in particular have a greater motivational salience for at-risk groups compared to control participants.

At the same time, the at-risk groups showed smaller increases in response to unpleasant images with high motivational salience (i.e., mutilation images). Although we predicted that the at-risk groups would have increased responses as the motivational salience of images increased, these results are consistent with the model of PRotective Inhibition of Self-regulation and Motivation (PRISM)^47^, which relates personality characteristics to dynamics within neural systems. Specifically, the PRISM model argues that “absorption,” which is akin to schizophrenia-like symptoms^48,49^, is related to greater physiological and emotional reactivity to emotional stimuli and to increased aberrant experiences when exposed to stressors. However, increasing intensity of stimuli, and subsequent neural responses, will protectively increase inhibition, thereby decreasing responses overall. Thus, it is possible that as the motivational salience of the unpleasant stimuli increased above a certain threshold, the at-risk groups showed a reduced neural response due to a protective mechanism. To investigate this possibility further, future research could examine whether electrophysiological responses to motivationally salience images differ for at-risk groups before and after an immediate stressor (e.g., the Trier Social Stress Test^50^) to test whether reactivity varies as a function of the exposure to the stressor.

Responses to neutral images were largely consistent across groups. These findings are consistent with results from a recent meta-analysis of P3/LPP amplitude differences in individuals with schizophrenia vs. controls. Specifically, Castro and colleagues^51^ found that across 13 studies (including 339 individuals with schizophrenia and 331 healthy controls), there was a very small, non-significant difference between the groups in response to neutral images (Hedges’ *g* = −0.06, 95% CI = −0.55, 0.43). This suggest that using neutral images as a “baseline” from which to compare groups’ responses to affective images can be an empirically-informed decision.

Although the current results are novel and have implications for our understanding of emotional abnormalities associated with schizophrenia-spectrum disorder risk, the study is not without limitations. One limitation involves the relatively smaller sample size retained for the late LPP analysis due to large number of trials required to achieve the recommended dependability cut off of 0.7^52^. This is particularly true for the Psychotic-like Experiences group, where 17 of the 25 participants were excluded due to low dependability. Although this analysis suggests our results are robust with the number of subjects retained, these findings should be interpreted with caution for the Psychotic-like Experiences group. Future research examining the late LPP component could aim for larger number of trials and/or sample size, especially for research on vulnerable populations. Another possible limitation involves the time in between each trial, which was jittered between 250 and 750 ms. Because the LPP can extend beyond 1000 ms post-stimuli onset, there is a potential contamination of previous trials’ LPPs on subsequent trials’ LPPs^31^. Although visual inspection of grand average waveforms from the current study suggests the LPP did not extend beyond 1500 ms, it is possible there was some contamination. Thus, future research could use extended inter-stimulus intervals (e.g., 2000 ms) to avoid any possibility of this. Also, other than our measures of schizophrenia-spectrum disorder risk, we did not include other measures of psychological functioning, such as current mood, depression or substance abuse, which can affect electrophysiological responses to emotional stimuli e.g.^53,54^. Thus, future research could include such measures to test whether they account for the current findings.

A final issue to consider is adjustments for family- and experiment-wise error inflation. In the current study, we did not make an adjustment for family-wise multiple comparisons because our use of multi-level models accounts for any issue of spurious results arising from testing our a priori hypotheses. In multi-level models, as were used for all analyses in the current study, the relationship between all parameters are specified in the model, and this allows for a more precise estimate of the relationship between such variables (see^55^ for further details). In addition, we did not correct for multiple comparisons in order to control the experiment-wise error rate because we did not predict that all groups would be different from each other in all conditions. Thus, as Perneger^56^ says, this would be an “irrelevant null hypothesis” and making adjustments in order to avoid a false rejection does not make theoretical sense^cf^^57^. However, the debate in science about when and how best to correct for family- and experiment-wise error inflation is ongoing e.g.^58,59^. Thus, all researchers should consider the best practices for correcting for false discoveries in their work.

Despite these limitations, the current study found unique patterns of responding between two groups of individuals at risk for developing schizophrenia-spectrum disorders by examining subcategories within broad valence categories. Overall, these findings suggest these two at-risk groups have both unique and shared abnormalities related to attention to images with increasing motivational salience. This study highlights the importance of investigating patterns of emotional responding beyond the broad “pleasant” and “unpleasant” categories as a way to identify specific dysfunctional emotion mechanisms.

## Methods and Materials

### Participants

Participants for this study were recruited from a group of individuals who completed the Wisconsin Schizotypy scales online (N = 2329). In addition to meeting criteria for either one of the at-risk group or the comparison group based on their scores on the Wisconsin Schizotypy scales (described below), participants were required to be a) 18 years of age or older, b) fluent in English, and c) right-hand dominant to be enrolled in the study. Individuals were not eligible for the study if they had a) any neurological illness or movement disorder (e.g., seizures, epilepsy, stroke, brain injury, Parkinson’s disease), b) a history of taking medication to change their mood, emotions or the way they thought or acted (e.g., mood stabilizers, anti-depressants, stimulants), c) an allergy to salt or latex, or d) non-removable piercings on their head or face. As part of the recruitment process, eligibility status was confirmed via a phone screen.

In the current study, there were 24 people in the Social Anhedonia group who scored 1.5 *SD* above the mean on the short version of the Revised Social Anhedonia Scale^60^. People with extremely elevated social anhedonia have been found to be at increased risk for schizophrenia-spectrum personality disorders (e.g., 24% at 10-year follow-up^8^). There were 27 people in the Psychotic-like Experiences group who scored 1.5 *SD* above the mean on the short versions of the Perceptual Aberration or Magical Ideation scales^60^. People with extremely elevated Perceptual Aberration/Magical Ideation scores have been found to be at increased risk for psychotic disorders^9^. There were 25 people in the Control group who, following previous research^9^, scored less than 0.5 *SD* above the mean on the short versions of the Revised Social Anhedonia Scale, Perceptual Aberration Scale, and Magical Ideation Scale.

Previous research has shown that participants with elevated scores on the Wisconsin Schizotypy scales, as were recruited here as our at-risk groups, report clinically meaningful psychotic-like experiences and anhedonia in an interview format (i.e., Structured Clinical Interview for Prodromal Symptoms^61^) and that these schizotypy scales are moderately to strongly correlated with interview-rated symptoms^62^. Thus, although a clinical interview was not conducted in the current study, evidence suggests that there would be a high correspondence between these self-report measures and interview ratings. This, taken together with previous longitudinal evidence e.g.^8,9^, indicates that the current study’s groups of extreme scorers do have an increased risk for schizophrenia-spectrum disorders and psychosis and are thus considered “at risk” for the development of a schizophrenia-spectrum disorder.

Due to a computer malfunction, no EEG data were saved for two subjects (Psychotic-like Experiences group *n* = 1; Control group *n* = 1) and, thus, no data were available from these subjects for analysis. Additionally, as discussed below, six participants were excluded from all analyses due to poor data quality (Social Anhedonia group *n* = 3; Psychotic-like Experiences group *n* = 1; Control group *n* = 2). The final sample included in the analyses consisted of 21 in the Social Anhedonia group, 25 in the Psychotic-like Experiences group, and 22 in the Control group. As can be seen in Table 3, there were no significant between-group differences on any demographic variable we assessed. However, there was a trend for the groups to differ with respect to gender composition. Because of this trend, coupled with data that suggests there are gender differences in self-reported experiences in response to affective images used in the current study^63^, we included gender as a covariate in our statistical analyses.

**Table 3:** Demographic Information by group.

	Control (*n* = 22)	Social Anhedonia (*n* = 21)	Psychotic-like Experiences (*n* = 25)	Group comparisons
Age (mean (*SD*))	20.91 (3.53)	20.10 (2.51)	19.36 (1.66)	*F*(2, 65) = 2.01, *p* = 0.14
Race				*p* = 0.21, two-tailed Fisher’s exact test
% African-American	0	9.52	0	
% Asian	40.91	28.57	56.00	
% Caucasian	22.73	9.52	20.00	
% Hispanic	31.82	33.33	20.00	
% Other	4.54	19.05	4.00	
Sex (% female)	77.27	90.48	64.0	*p* = 0.10, two-tailed Fisher’s exact test
Short Revised Social Anhedonia Scale (mean (*SD*))	1.14 (0.77)	7.24 (1.61)	1.84 (1.43)	*F* (2, 65) = 137.7, *p* < 0.001 Control, PLEs < SocAnh
Short Perceptual Aberration Scale (mean (*SD*))	0.86 (0.71)	1.33 (1.56)	6.72 (3.77)	*F* (2, 65) = 40.83, *p* < 0.001 Control, SocAnh < PLEs
Short Magical Ideation Scale (mean (*SD*))	2.36 (1.00)	1.90 (1.95)	8.36 (3.16)	*F* (2, 65) = 59.27, *p* < 0.001 Control, SocAnh < PLEs

Note: Participants were included in this table if they had any ERP data included in the ERP analyses.

### Materials

#### Wisconsin Schizotypy scales

Participants completed the short versions of the Wisconsin Schizotypy scales^60^. These include the short version of the Revised Social Anhedonia Scale (α in current study = 0.78; for all participants, *M* = 3.26, *SD* = 2.97), which is designed to measure lack of relationships and lack of pleasure from relationships (e.g., “Having close friends is not as important as many people say”). They also completed the short version of the Perceptual Aberration Scale (α in current study = 0.88; for all participants, *M* = 3.11, *SD* = 3.60) and the short version of the Magical Ideation Scale (α in current study = 0.85; for all participants, *M* = 4.30, *SD* = 3.76), which are designed to measure psychotic-like distortions and unusual beliefs, respectively (e.g., “I have sometimes had the feeling that one of my arms or legs is disconnected from the rest of my body”; “Some people can make me aware of them by just thinking about me”). Previous research has shown that the short versions of the Wisconsin Schizotypy scales have superior psychometric properties compared to the full versions, particularly in their measure of schizotypy in men vs. women^60^ and in ethnically-diverse samples^64^. Thus, the means used to determine group status were the same across demographic characteristics.

#### ERP paradigm

Following the procedure of Weinberg and Hajcak^25^, there were three trial blocks, with each block consisting of 45 pleasant (15 exciting, 15 affiliative, and 15 erotic images), 45 unpleasant (15 disgusting, 15 threatening, and 15 mutilation images), or 45 neutral images (15 object, 15 scenes without people, and 15 scenes with people). Both block order and trial order within blocks were randomized between participants. As discussed by Weinberg and Hajcak^25^, images were displayed in blocks to minimize effects of display probability and surprise.

Images in each subcategory were identical to the ones used by Weinberg and Hajcak^25^, which were selected from the International Affective Picture System (IAPS)^63^. The images significantly differed in normative valence (pleasant > neutral > unpleasant) and arousal ratings (pleasant = unpleasant > neutral) as expected. Based on theory e.g.^24^, normative ratings^63^, and previous research e.g.^25^, images within each broad category were classified as having “low”, “moderate” or “high” motivational significance (rank order of images’ motivational significance within each broad category from lowest to highest: Pleasant - exciting, affiliative, erotic; Unpleasant - disgusting, threatening, mutilation; Neutral - objects, scenes without people, scenes with people), and these categorizations were used in the statistical analyses described below. Please see the Supplementary Materials for a list of the IAPS images used in each subcategory.

In each trial of the task, participants were first shown a fixation cross for 2000 ms and then an image for 1500 ms. The inter-trial interval was jittered and ranged from 250–750 ms. A total of 270 trials were presented across three blocks. Within each block, participants viewed each image twice for a total of 90 trials per block. Participants were told to “respond naturally” to the image, which was explained to mean that they were free to experience any emotional response that may spontaneously occur when viewing the image and to make no attempt to alter this natural emotional response in any way. ERPs were recorded while participants passively viewed the images, and researchers who collected these data were blind to group membership at the time of collection.

#### Image ratings

After EEG recording was completed, participants were asked to rate the valence (*extremely positive* to *extremely negative*) and arousal levels (*no bodily response* to *strong bodily response*) of a random subgroup of images (*n* = 30, 10 randomly selected from each broad category) that they previously viewed, using 1–9 scales. Participants were not asked to rate of all the images they viewed during the ERP paradigm to minimize participant burden (e.g., limiting the number of times they viewed extremely negative images).

### Procedure

This study, which was approved by the University’s IRB, was carried out in accordance with the latest version of the Declaration of Helsinki. All participants provided informed consent.

Participants were seated alone in a quiet, temperature-controlled room. After electrode placement, they completed both the passive image viewing task described above and another, unrelated task not reported here in counterbalanced order. Immediately following the EEG session, participants made valence and arousal ratings of images from the ERP task.

### EEG recording and processing

The EEG was recorded from 32 sintered Ag/AgCl electrodes (FP1, FPz, FP2, Fz, F3, F4, F7, F8, FC5, FC1, FC2, FC6, Cz, C3, C4, T7, T8, CP1, CP2, CP5, CP6, Pz, P3, P4, P7, P8, POz, Oz, O1, O2, M1, M2) fixed in an electrode cap (ANT Neuro, ﻿Enschede, The Netherlands) and placed according to an expanded 10/20 system^65^. All EEG electrodes were referenced online to CPz using a sampling rate of 1000 Hz; an average mastoid reference was derived offline. EEG was amplified with an eego sports amplifier (ANT Neuro, ﻿Enschede, The Netherlands). No online filter was used. Impedance was kept below 5kΩ at all electrodes.

All signal processing and analysis procedures were conducted through EEGlab^66^ with the ERPLAB toolbox^67^ for MATLAB^68^. Data were first downsampled to 500 Hz and a Butterworth highpass filter of 0.1 Hz was used. Then, large muscle artifacts or extreme offsets were identified and removed through a semi-automated procedure that identified voltage deflections of ±200 microvolts (µV). Identification and removal of eye-blink artifacts was accomplished using an independent component analysis (ICA). After the ICA, stimulus-locked epochs of 1700 ms (including 200 ms pre-stimulus baseline) were created for each channel. Next, a peak-to-peak artifact rejection procedure was used that involved a moving window of 200 ms and a window step of 50 ms to remove epochs containing ± 100 microvolts (µV) deflection in any of the channels that were to be used for ERP analyses (EPN: POz, Oz, O1, O2; LPP: Pz, P3, P4, CPz, CP1, CP2). Finally, data were filtered with a Butterworth lowpass, half-amplitude filter of 30 Hz.

Following recommendations of prior research^52^, reliability of each ERP component of interest was quantified by generalization theory’s index of dependability and calculated using the ERP Reliability Analysis (ERA) toolbox^69^. As can be seen in Table 4, participants with fewer trials to achieve a dependability estimate of 0.7 were excluded. Based on these results, we included participants’ artifact-free data in group grand average waveforms for each component.

**Table 4:** Psychometric properties of ERP components by group.

	Control	Social Anhedonia	Psychotic-like Experiences	Group comparisons
Minimum number of trials^a^				
EPN	12	7	8	χ^2^(2) = 1.56, *p* = 0.46
Early LPP	22	20	33	χ^2^(2) = 3.92, *p* = 0.14
Late LPP	67	70	126	χ^2^(2) = 25.19, *p* < 0.001 Control, SocAnh <PLEs
Number of participants excluded				
EPN	2	1	1	χ^2^(2) = 0.5, *p* = 0.78
Early LPP	5	2	3	χ^2^(2) = 1.4, *p* = 0.50
Late LPP	9	7	17	χ^2^(2) = 5.09, *p* = 0.08
Dependability (95% CI)				
EPN	0.95 [0.91 0.97]	0.97 [0.96 0.99]	0.97 [0.95 0.99]	*p* > 0.05
Early LPP	0.92 [0.85 0.96]	0.94 [0.89 0.98]	0.90 [0.83 0.95]	*p* > 0.05
Late LPP	0.81 [0.62 0.92]	0.85 [0.70 0.94]	0.78 [0.59 0.91]	*p* > 0.05

Note: EPN = Early Posterior Negativity; LPP = Late Positive Potential; CI = confidence interval

^a^Denotes minimum number of trials to reach the dependability point estimate of > 0.70.

### Analyses

#### Power analysis

A power analysis was conducted using the package SIMR^70^ for R^71^. SIMR uses Monte Carlo simulations to determine power estimates for models that include both fixed and random effects. Using the data and effect sizes reported in Martin and colleagues^16^ (an ERP study of at-risk individuals), it was determined that, based on 1000 simulations, an alpha level of 0.05, and a sample size of 20 per group, the power to detect an effect of similar size was greater than 0.99. Thus, we aimed to recruit 22 subjects per group, which would allow for some missing data after ERP data reduction.

#### ERP statistical analyses

Previous research found that the EPN is largest at occipital electrodes around 240 ms^25,26,30^, and visual inspection of grand average waveforms across all participants found this to be true in the current study. Thus, consistent with previous research, the EPN was scored as the average activity at POz, Oz, O1, and O2 between 200 and 280 ms.

Previous research has found the LPP to be maximal in the centro-parietal region^16,25,33,72–74^, and visual inspection of grand averages across all participants found this to be true in the current study. Thus, consistent with previous research, the LPP amplitude was scored as the average amplitude from six centro-parietal sites (Pz, P3, P4, CPz, CP1, CP2). Also consistent with previous research^25,73,75^, the LPP was divided into early and late portions, defined as the average voltage occurring between 400–1000 and 1000–1500 ms post-stimulus, respectively.

As in many other ERP studies e.g.^76–79^, we used mixed hierarchical linear models (HLMs) to test the effects of the stimuli on ERP amplitudes across groups. When analyzing psychophysiological data, multivariate approaches, such as HLM, have several advantages over univariate approaches^80,81^. Briefly, multivariate approaches do not make the same statistical assumptions often required by univariate models (e.g., sphericity^82^), and therefore do not require corrections for violations of these assumptions that result in reduced statistical power. Also, the within-subject variability in EEG data is often greater than the variability associated with manipulated variables of interest^80^, contributing to inflated error variance in univariate models that can also reduce statistical power. The use of an intercept for each electrode within each subject, as used in the current statistical approach, reduces these error variance estimates, thereby maintaining power. In addition, multivariate approaches do not use listwise deletion for instances of missing data, and therefore are more robust to incomplete data across individuals (e.g., artifact rejection in EEG resulting in different numbers of electrodes per subject in any given condition).

All statistical analyses were conducted using R^71^. Specific packages used to run HLM models in R included nlme^83^, lme4^84^, and lmerTest^85^. For each ERP component of interest, we ran a mixed model for each broad valence category (pleasant, unpleasant, neutral) to test how differences in amplitudes varied as the motivational salience of images increased: Group (Control vs. Social Anhedonia vs. Psychotic-like Experiences) X Subcategory Image Type (Low motivational salience vs. Moderate motivational salience vs. High motivational salience). All models included random intercepts of subject and of electrodes within subjects as well as gender as a covariate.

There is no agreed upon measure to indicate the size of effects in HLMs e.g.^86^. However, a widely used index is the “partially standardized coefficient (β_partial_)”, which is calculated by standardizing the outcome variable and using dummy or effect coded predictors^87^. They are named “partially” standardized coefficients because only the outcome variables, but not the predictors, were standardized. We did not use “fully” standardized coefficients given that all of our predictors are categorical variables (e.g., group). Thus, standardizing the predictors would not produce meaningful parameter estimates. In the current study, β_partial_ represents differences in standard deviation units, rather than raw units, between groups.

#### Image rating analyses

For valence and arousal ratings of images, we ran separate linear mixed model of Group (Control vs. Social Anhedonia vs. Psychotic-like Experiences) X Subcategory Image Type (Low vs. Moderate vs. High motivational salience) within each broad category and included random intercepts of subject as well as gender as a covariate.

## Supplementary information

Supplemental information.

## Data Availability

The dataset generated during the current study are available from the corresponding author on reasonable request.

## References

[1] Lang, P. J., Bradley, M. M. & Cuthbert, B. N. Motivated attention: Affect, activation, and action. in *Attention and orienting: Sensory and motivational processes* (eds. Lang, P. J., Simons, R. F. & Balaban, M. T.) 97–135 (Lawrence Erlbaum Associates Publishers, 1997).

[2] Schultz W (2016). Dopaminze reward prediction-error signalling: A two-component response. Nat. Rev. Neurosci..

[3] Cohen AS, Minor KS (2010). Emotional experience in patients with schizophrenia revisited: Meta-analysis of laboratory studies. Schizophr. Bull..

[4] Kohler CG, Martin EA (2006). Emotional processing in schizophrenia. Cogn. Neuropsychiatry.

[5] Green MF, Hellemann G, Horan WP, Lee J, Wynn JK (2012). From perception to functional outcome in schizophrenia: Modeling the role of ability and motivation. Arch. Gen. Psychiatry.

[6] Kring AM, Gur RE, Blanchard JJ, Horan WP, Reise SP (2013). The Clinical Assessment Interview for Negative Symptoms (CAINS): Final development and validation. Am. J. Psychiatry.

[7] Gooding DC, Tallent KA, Matts CW (2005). Clinical status of at-risk individuals 5 years later: Further validation of the psychometric high-risk strategy. J. Abnorm. Psychol..

[8] Kwapil TR (1998). Social anhedonia as a predictor of the development of schizophrenia- spectrum disorders. J. Abnorm. Psychol..

[9] Chapman LJ, Chapman JP, Kwapil TR, Eckblad M, Zinser MC (1994). Putatively psychosis-prone subjects 10 years later. J. Abnorm. Psychol..

[10] Brown LH, Silvia PJ, Myin-Germeys I, Kwapil TR (2007). When the need to belong goes wrong: The expression of social anhedonia and social anxiety in daily life. Psychol. Sci..

[11] Kerns JG, Docherty AR, Martin EA (2008). Social and physical anhedonia and valence and arousal aspects of emotional experience. J. Abnorm. Psychol..

[12] Martin EA, Becker TM, Cicero DC, Docherty AR, Kerns JG (2011). Differential associations between schizotypy facets and emotion traits. Psychiatry Res..

[13] Martin EA, Becker TM, Cicero DC, Kerns JG (2013). Examination of affective and cognitive interference in schizophrenia and relation to symptoms. J. Abnorm. Psychol..

[14] Martin EA, Cicero DC, Bailey DH, Karcher NR, Kerns JG (2016). Social anhedonia is not just extreme introversion: Empirical evidence of distinct constructs. J. Pers. Disord..

[15] Martin, E. A., Hua, J. P. Y., Straub, K. T. & Kerns, J. G. Explicit and implicit affect and judgment in schizotypy. *Front. Psychol*. **10**, (2019). 10.3389/fpsyg.2019.01491 PMC6613436 31312158

[16] Martin EA, Karcher NR, Bartholow BD, Siegle GJ, Kerns JG (2017). An electrophysiological investigation of emotional abnormalities in groups at risk for schizophrenia-spectrum personality disorders. Biol. Psychol..

[17] Karcher N, Shean G (2012). Magical ideation, schizotypy and the impact of emotions. Psychiatry Res..

[18] Cicero DC, Becker TM, Martin EA, Docherty AR, Kerns JG (2013). The role of aberrant salience and self-concept clarity in psychotic-like experiences. Personal. Disord. Theory, Res. Treat..

[19] Neumann SR, Linscott RJ (2018). The relationships among aberrant salience, reward motivation, and reward sensitivity. Int. J. Methods Psychiatr. Res..

[20] Gooding DC, Davidson RJ, Putnam KM, Tallent KA (2002). Normative emotion-modulated startle response in individuals at risk for schizophrenia–spectrum disorders. Schizophr. Res..

[21] Martin EA, Kerns JG (2010). Social anhedonia associated with poor evaluative processing but not with poor cognitive control. Psychiatry Res..

[22] Strauss GP, Ruiz I, Visser KH, Crespo LP, Dickinson EK (2018). Diminished Hedonic response in neuroleptic-free youth at ultra high-risk for psychosis. Schizophr. Res. Cogn..

[23] Yang Yin, Yang Zhuo-ya, Zou Ying-min, Shi Hai-song, Wang Yi, Xie Dong-jie, Zhang Rui-ting, Lui Simon S.Y., Cohen Alex C., Strauss Gregory P., Cheung Eric F.C., Chan Raymond C.K. (2018). Low-pleasure beliefs in patients with schizophrenia and individuals with social anhedonia. Schizophrenia Research.

[24] Bradley MM, Codispoti M, Cuthbert BN, Lang PJ (2001). Emotion and motivation I: Defensive and appetitive reactions in picture processing. Emotion.

[25] Weinberg A, Hajcak G (2010). Beyond good and evil: The time-course of neural activity elicited by specific picture content. Emotion.

[26] Foti D, Hajcak G, Dien J (2009). Differentiating neural responses to emotional pictures: Evidence from temporal-spatial PCA. Psychophysiology.

[27] Hajcak G, MacNamara A, Olvet DM (2010). Event-related potentials, emotion, and emotion regulation: An integrative review. Dev. Neuropsychol..

[28] Schupp HT, Junghöfer M, Weike AI, Hamm AO (2003). Attention and emotion: An ERP analysis of facilitated emotional stimulus processing. Neuroreport.

[29] Cuthbert BN, Schupp HT, Bradley MM, Birbaumer N, Lang PJ (2000). Brain potentials in affective picture processing: Covariation with autonomic arousal and affective report. Biol. Psychol..

[30] Dunning JP (2011). Motivated attention to cocaine and emotional cues in abstinent and current cocaine users - an ERP study. Eur. J. Neurosci..

[31] Hajcak G, Olvet DM (2008). The persistence of attention to emotion: Brain potentials during and after picture presentation. Emotion.

[32] Luck, S. J. *An introduction to the event-related potential technique*. doi:9780262525855 (MIT press, 2014).

[33] Schupp HT (2000). Affective picture processing: The late positive potential is modulated by motivational relevance. Psychophysiology.

[34] Schupp HT (2004). Brain processes in emotional perception: Motivated attention. Cogn. Emot..

[35] Martin EA, Cicero DC, Kerns JG (2012). Social anhedonia, but not positive schizotypy, is associated with poor affective control. Personal. Disord. Theory, Res. Treat..

[36] Martin Elizabeth A., Siegle Greg J., Steinhauer Stuart R., Condray Ruth (2019). Timing matters in elaborative processing of positive stimuli: Gamma band reactivity in schizophrenia compared to depression and healthy adults. Schizophrenia Research.

[37] Applegate E, El-Deredy W, Bentall RP (2009). Reward responsiveness in psychosis-prone groups: Hypomania and negative schizotypy. Pers. Individ. Dif..

[38] Mathews A, MacLeod C (2005). Cognitive vulnerability to emotional disorders. Annu. Rev. Clin. Psychol..

[39] Price RB (2015). Empirical recommendations for improving the stability of the dot-probe task in clinical research. Psychol. Assess..

[40] MacLeod C, Rutherford E, Campbell L, Ebsworthy G, Holker L (2002). Selective attention and emotional vulnerability: Assessing the causal basis of their association through the experimental manipulation of attentional bias. J. Abnorm. Psychol..

[41] MacLeod C, Mathews A (2012). Cognitive bias modification approaches to anxiety. Annu. Rev. Clin. Psychol..

[42] Chapman, L. J., Chapman, J. P., Raulin, M. L. & Edell, W. S. *Schizotypy and thought disorder as a high risk approach to schizophrenia*. (Brunner/Mazel, 1978).

[43] Eckblad M, Chapman LJ (1983). Magical ideation as an indicator of schizotypy. J. Consult. Clin. Psychol..

[44] Alloy LB, Olino T, Freed RD, Nusslock R (2016). Role of reward sensitivity and processing in major depressive and bipolar spectrum disorders. Behav. Ther..

[45] Cicero DC, Kerns JG (2010). Multidimensional factor structure of positive schizotypy. J. Pers. Disord..

[46] Kwapil TR, Brown LH, Barrantes-Vidal N (2010). P03-26 - Paranoia, schizotypy, and social anxiety: Factor structure and experience in daily life. Eur. Psychiatry.

[47] Tops, M., Montero-Marin, J. & Quirin, M. Too much of a good thing: A neuro-dynamic personality model explaining engagement and its protective inhibition. in *Recent Developments in Neuroscience Research on Human Motivation* (eds. Kim, S., Reeve, J. & Bong, M.) **19**, 283–319 (Emerald Group Publishing Limited, 2016).

[48] Lange R, Thalbourne MA, Houran J, Storm L (2000). The Revised Transliminality Scale: Reliability and validity data from a Rasch top-down purification procedure. Conscious. Cogn..

[49] Parker A (1999). Imaginal experiences and perceptual defence. Br. J. Med. Psychol..

[50] Kirschbaum C, Pirke KM, Hellhammer DH (1993). The ‘Trier Social Stress Test’ - A tool for investigating psychobiological stress responses in a laboratory setting. Neuropsychobiology.

[51] Castro MK, Bailey DH, Zinger JF, Martin EA (2019). Late electrophysiological potentials and emotion in schizophrenia: A meta-analytic review. Schizophr. Res..

[52] Clayson PE, Miller GA (2017). Psychometric considerations in the measurement of event-related brain potentials: Guidelines for measurement and reporting. Int. J. Psychophysiol..

[53] Bartholow BD, Pearson MA, Gratton G, Fabiani M (2003). Effects of alcohol on person perception: A social cognitive neuroscience approach. J. Pers. Soc. Psychol..

[54] Hill KE, South SC, Egan RP, Foti D (2019). Abnormal emotional reactivity in depression: Contrasting theoretical models using neurophysiological data. Biol. Psychol..

[55] Gelman A, Hill J, Yajima M (2012). Why we (usually) don’t have to worry about multiple comparisons. J. Res. Educ. Eff..

[56] Perneger TV (1998). What’s wrong with Bonferroni adjustments. BMJ.

[57] Aickin M (1999). Other method for adjustment of multiple testing exists. BMJ.

[58] Jafari M, Ansari-Pour N (2019). Why, when and how to adjust your P values?. Cell J..

[59] Glickman ME, Rao SR, Schultz MR (2014). False discovery rate control is a recommended alternative to Bonferroni-type adjustments in health studies. J. Clin. Epidemiol..

[60] Winterstein BP (2011). Brief assessment of schizotypy: Developing short forms of the Wisconsin Schizotypy Scales. Pers. Individ. Dif..

[61] Miller TJ (2003). Prodromal assessment with the Structured Interview for Prodromal Syndromes and the Scale of Prodromal Symptoms: Predictive validity, interrater reliability, and training to reliability. Schizophr. Bull..

[62] Cicero DC, Martin EA, Becker TM, Docherty AR, Kerns JG (2014). Correspondence between psychometric and clinical high risk for psychosis in an undergraduate population. Psychol. Assess..

[63] Acharya Naresh, Mithulananthan N. (2007). Locating series FACTS devices for congestion management in deregulated electricity markets. Electric Power Systems Research.

[64] Cicero DC, Martin EA, Krieg A (2019). Differential item functioning of the full and brief Wisconsin Schizotypy Scales in Asian, White, Hispanic, and multiethnic samples. Assessment.

[65] American Electroencephalographic Society (1994). Guideline thirteen: Guidelines for standard electrode position nomenclature. J. Clin. Neurophysiol..

[66] Delorme A, Makeig S (2004). EEGLAB: An open source toolbox for analysis of single-trial EEG dynamics including independent component analysis. J. Neurosci. Methods.

[67] Lopez-Calderon J, Luck SJ (2014). ERPLAB: An open-source toolbox for the analysis of event-related potentials. Front. Hum. Neurosci..

[68] The MathWorks Inc. MATLAB and Statistics Toolbox Release 2018b. (2018).

[69] Clayson PE, Miller GA (2017). ERP Reliability Analysis (ERA) Toolbox: An open-source toolbox for analyzing the reliability of event-related brain potentials. Int. J. Psychophysiol..

[70] Green P, MacLeod CJ (2016). SIMR: an R package for power analysis of generalized linear mixed models by simulation. Methods Ecol. Evol..

[71] R Core Team. R: A language and environment for statistical computing. (2017).

[72] Foti D, Hajcak G (2008). Deconstructing reappraisal: Descriptions preceding arousing pictures modulate the subsequent neural response. J. Cogn. Neurosci..

[73] Hajcak G, Dunning JP, Foti D (2007). Neural response to emotional pictures is unaffected by concurrent task difficulty: An event-related potential study. Behav. Neurosci..

[74] Keil A (2002). Large-scale neural correlates of affective picture processing. Psychophysiology.

[75] Dennis TA, Hajcak G (2009). The late positive potential: A neurophysiological marker for emotion regulation in children. J. Child Psychol. Psychiatry Allied Discip..

[76] Brush CJ, Ehmann PJ, Hajcak G, Selby EA, Alderman BL (2018). Using multilevel modeling to examine blunted neural responses to reward in major depression. Biol. Psychiatry Cogn. Neurosci. Neuroimaging.

[77] Hilgard J, Weinberg A, Hajcak Proudfit G, Bartholow BD (2014). The negativity bias in affective picture processing depends on top-down and bottom-up motivational significance. Emotion.

[78] Tremblay A, Newman AJ (2015). Modeling nonlinear relationships in ERP data using mixed-effects regression with R examples. Psychophysiology.

[79] Wierda SM, van Rijn H, Taatgen NA, Martens S (2010). Distracting the mind improves performance: An ERP study. PLoS One.

[80] Gratton, G. Biosignal processing. in *Handbook of psychophysiology* (eds. Cacioppo, J. T., Tassinary, L. G. & Bernston, G. G.) (Cambridge University Press, 2007).

[81] Vasey MW, Thayer JF (1987). The continuing problem of false positives in repeated measures ANOVA in psychophysiology: A multivariate solution. Psychophysiology.

[82] Jennings RJ, Wood CC (1976). Letter: The epsilon-adjustment procedure for repeated-measures analyses of variance. Psychophysiology.

[83] Pinheiro, J., Bates, D., DebRoy, S., Sarkar, D. & R Core Team. nlme: Linear and nonlinear mixed effects models. R package version 3.1-141. (2018).

[84] Bates, D., Mächler, M., Bolker, B. & Walker, S. Fitting linear mixed-effects models using lme4o. *J. Stat. Softw*. **67**, (2015).

[85] Kuznetsova, A., Brockhoff, P. B. & Christensen, R. H. B. lmerTest package: Tests in linear mixed effects models. *J. Stat. Softw*. **82**, (2017).

[86] Peugh JL (2010). A practical guide to multilevel modeling. J. Sch. Psychol..

[87] Lorah J (2018). Effect size measures for multilevel models: Definition, interpretation, and TIMSS example. Large-scale Assessments Educ..

