# Supplementary material for: Electrophysiological responses to images ranging in motivational salience: Attentional abnormalities associated with schizophrenia-spectrum disorder risk
Source: Sci Rep. 2020 Mar 12;10:4578. doi: 10.1038/s41598-020-61504-2 (PMC7067785; doi:10.1038/s41598-020-61504-2)
Supplement: Supplementary file 1 — Supplemental information. [file 41598_2020_61504_MOESM1_ESM.docx]

| Supplementary Table 1  *Estimates of Fixed Effects for Models Predicting EPN amplitudes*  *____________________________________________________________________________* | | | | |
| --- | --- | --- | --- | --- |
|  | | | | |
|  |  | Partially standardized  estimate (SE) | *t* | *p* |
| Model 1: Pleasant Valence only^1^ | |  |  |  |
|  |  |  |  |  |
| Controls vs. SocAnh |  |  |  |  |
|  | Affiliative | 0.11 (0.16) | 0.66 | .51 |
|  | Erotic | 0.33 (0.16) | 2.08 | .03* |
| Controls vs. Psychotic-like Experiences |  |  |  |  |
|  | Affiliative | -0.04 (0.15) | -0.29 | .77 |
|  | Erotic | 0.11 (0.15) | 0.75 | .45 |
| Psychotic-like Experiences vs. SocAnh |  |  |  |  |
|  | Affiliative | 0.15 (0.15) | 0.98 | .33 |
|  | Erotic | 0.22 (0.15) | 1.43 | .15 |
|  |  |  |  |  |
| Model 3: Unpleasant Valence only^2^ | |  |  |  |
| Controls vs. SocAnh |  |  |  |  |
|  | Threatening | 0.019 (.15) | 1.28 | .20 |
|  | Mutilation | 0.0004 (.15) | 0.003 | .99 |
| Controls vs. Psychotic-like Experiences |  |  |  |  |
|  | Threatening | 0.036 (.14) | 0.26 | .79 |
|  | Mutilation | -0.008 (.13) | -0.61 | .54 |
| Psychotic-like Experiences vs. SocAnh |  |  |  |  |
|  | Threatening | 0.15 (0.14) | 1.08 | .28 |
|  | Mutilation | 0.08 (0.15) | 0.57 | .57 |
|  |  |  |  |  |
| Model 3: Neutral Valence only^3^ | |  |  |  |
| Controls vs. SocAnh |  |  |  |  |
|  | Scenes without people | -0.14(0.12) | -1.14 | .25 |
|  | Scenes with people | -0.12 (0.12) | -0.98 | .32 |
| Controls vs. Psychotic-like Experiences |  |  |  |  |
|  | Scenes without people | -0.02 (0.12) | -0.18 | .86 |
|  | Scenes with people | -0.21 (0.12) | -1.80 | .08 |
| Psychotic-like Experiences vs. SocAnh |  |  |  |  |
|  | Scenes without people | -0.12 (0.13) | -0.88 | .38 |
|  | Scenes with people | 0.09 (0.13) | 0.65 | .52 |

Note: * *p* < 0.05; ** *p* < 0.01; *** *p* < 0.001. The first group listed in the first column is the reference group. ^1^The Exciting images subcategory is the reference subcategory. ^2^The Disgusting images subcategory is the reference subcategory. ^3^The Object images subcategory is the reference subcategory.

| Supplementary Table 2  *Estimates of Fixed Effects for Models Predicting Early LPP amplitudes*  *____________________________________________________________________________* | | | | |
| --- | --- | --- | --- | --- |
|  | | | | |
|  |  | Partially standardized  estimate (SE) | *t* | *p* |
| Model 1: Pleasant Valence only^1^ | |  |  |  |
|  |  |  |  |  |
| Controls vs. SocAnh |  |  |  |  |
|  | Affiliative images | 0.06 (0.14) | 0.40 | .69 |
|  | Erotic images | 0.05 (0.14) | 0.38 | .70 |
| Controls vs. Psychotic-like Experiences |  |  |  |  |
|  | Affiliative images | 0.20 (0.14) | 1.49 | .14 |
|  | Erotic images | 0.38 (0.14) | 2.73 | .007** |
| Psychotic-like Experiences vs. SocAnh |  |  |  |  |
|  | Affiliative images | -0.15 (0.14) | -1.08 | .28 |
|  | Erotic images | 0.32 (0.14) | 2.36 | .02* |
|  |  |  |  |  |
| Model 3: Unpleasant Valence only^2^ | |  |  |  |
| Controls vs. SocAnh |  |  |  |  |
|  | Threatening images | 0.70 (0.14) | 5.08 | < .001*** |
|  | Mutilation images | -0.20 (0.14) | -1.44 | .15 |
| Controls vs. Psychotic-like Experiences |  |  |  |  |
|  | Threatening images | 0.36 (0.13) | 2.79 | .005** |
|  | Mutilation images | -0.15 (0.13) | -1.16 | .25 |
| Psychotic-like Experiences vs. SocAnh |  |  |  |  |
|  | Threatening images | 0.34 (0.13) | 2.59 | .009** |
|  | Mutilation images | -0.05 (0.13) | -0.38 | .70 |
|  |  |  |  |  |
| Model 3: Neutral Valence only^3^ | |  |  |  |
| Controls vs. SocAnh |  |  |  |  |
|  | Scenes without people | -0.03 (.15) | -0.22 | .82 |
|  | Scenes with people | -0.02 (.15) | -0.11 | .92 |
| Controls vs. Psychotic-like Experiences |  |  |  |  |
|  | Scenes without people | -0.25 (.14) | -1.74 | .08 |
|  | Scenes with people | -0.20 (.14) | -1.42 | .15 |
| Psychotic-like Experiences vs. SocAnh |  |  |  |  |
|  | Scenes without people | 0.21 (.15) | 1.43 | .15 |
|  | Scenes with people | 0.18 (.15) | 1.25 | .21 |
|  |  |  |  |  |

Note: * *p* < 0.05; ** *p* < 0.01; *** *p* < 0.001. The first group listed in the first column is the reference group. ^1^The Exciting images subcategory is the reference subcategory. ^2^The Disgusting images subcategory is the reference subcategory. ^3^The Object images subcategory is the reference subcategory.

| Supplementary Table 3  *Estimates of Fixed Effects for Models Predicting Late LPP amplitudes*  *____________________________________________________________________________* | | | | |
| --- | --- | --- | --- | --- |
|  | | | | |
|  |  | Partially standardized  estimate (SE) | *t* | *p* |
| Model 1: Pleasant Valence only^1^ | |  |  |  |
|  |  |  |  |  |
| Controls vs. SocAnh |  |  |  |  |
|  | Affiliative images | -0.33 (0.15) | -2.25 | .02* |
|  | Erotic images | -0.44 (0.15) | -2.99 | .003** |
| Controls vs. Psychotic-like Experiences |  |  |  |  |
|  | Affiliative images | -.23 (0.16) | -1.43 | .15 |
|  | Erotic images | -0.03 (0.16) | -0.20 | .84 |
| Psychotic-like Experiences vs. SocAnh |  |  |  |  |
|  | Affiliative images | -0.10 (0.16) | -0.62 | .54 |
|  | Erotic images | -0.41 (0.16) | -2.53 | .01* |
|  |  |  |  |  |
| Model 3: Unpleasant Valence only^2^ | |  |  |  |
| Controls vs. SocAnh |  |  |  |  |
|  | Threatening images | 0.35 (0.15) | 2.26 | .02* |
|  | Mutilation images | -0.70 (0.15) | -4.57 | < .001*** |
| Controls vs. Psychotic-like Experiences |  |  |  |  |
|  | Threatening images | -0.12 (0.17) | -0.69 | .49 |
|  | Mutilation images | -0.54 (0.17) | -3.21 | .001** |
| Psychotic-like Experiences vs. SocAnh |  |  |  |  |
|  | Threatening images | 0.46 (0.17) | 2.71 | .006** |
|  | Mutilation images | -0.16 (0.17) | -0.94 | .34 |
|  |  |  |  |  |
| Model 3: Neutral Valence only^3^ | |  |  |  |
| Controls vs. SocAnh |  |  |  |  |
|  | Scenes without people | -0.17 (0.17) | -0.96 | .34 |
|  | Scenes with people | -0.12 (0.17) | -0.07 | .94 |
| Controls vs. Psychotic-like Experiences |  |  |  |  |
|  | Scenes without people | -0.55 (0.18) | -2.98 | .003** |
|  | Scenes with people | -0.004 (0.18) | -0.02 | .98 |
| Psychotic-like Experiences vs. SocAnh |  |  |  |  |
|  | Scenes without people | 0.38 (0.19) | 2.00 | .05 |
|  | Scenes with people | -0.01 (0.19) | -0.04 | .97 |

Note: * *p* < 0.05; ** *p* < 0.01; *** *p* < 0.001. The first group listed in the first column is the reference group. ^1^The Exciting images subcategory is the reference subcategory. ^2^The Disgusting images subcategory is the reference subcategory. ^3^The Object images subcategory is the reference subcategories.

**Supplementary Image Rating Results**

**Pleasant images**

Using exciting images as a reference, affiliative images were rated as more positive/less negative (β_partial_ = -0.62, *t*_128_ = -2.46, *p* = .02) and erotic images were rated as less positive/more negative (β_partial_ = 0.55, *t*_128_ = 2.18, *p* = .03) for the Control group. At-risk groups did not significantly differ from the Control group, or with each other, in their valence ratings of exciting, affiliative, or erotic images (all *p*s > .25). With respect to arousal, erotic (β_partial_ = 0.48, *t*_128_ = 2.21, *p* = .03), but not affiliative (β_partial_ = 0.21, *t*_128_ = 0.96, *p* = .34), images were rated as significantly more arousing than exciting images for the Control group. At-risk groups did not significantly differ from the Control group, or with each other, in their arousal ratings of exciting, affiliative, or erotic images (all *p*s > .14).

**Unpleasant images**

Neither threatening (β_partial_ = -0.31, *t*_128_ = -1.54, *p* = .13) nor mutilation images (β_partial_ = 0.31, *t*_128_ = 1.54, *p* = .13) significantly differed in valence ratings from disgusting images for the Control group. At-risk groups did not significantly differ from the Control group, or with each other, in their valence ratings of disgusting, threatening, or mutilation images (all *p*s > .41). With respect to arousal, threatening images were rated as less arousing than disgusting images for the Control group (β_partial_ = -0.53, *t*_128_ = -2.68, *p* = .008), while ratings for mutilation and disgusting images did not significantly differ (β_partial_ = 0.16, *t*_128_ = 0.79, *p* = .43). The Social Anhedonia group gave lower arousal ratings for disgusting images relative to both the Control group (β_partial_ = -0.68, *t*_117_ = -2.29, *p* = .02) and the Psychotic-like Experiences group (β_partial_ = -0.61, *t*_115_ = -2.09, *p* = .04). The Psychotic-like Experiences group gave trend-level greater arousal ratings for threatening images compared to the Control group (β_partial_ = 0.47, *t*_128_ = 1.75, *p* = .08).

**Neutral images**

Neither scenes without people (β_partial_ = 0.45, *t*_122_ = 1.66, *p* = .10) nor scenes with people (β_partial_ = -0.0006, *t*_122_ = -0.002, *p* = 1.00) significantly differed in valence ratings from images of objects for the Control group. Relative to the Control group, both the Social Anhedonia group (β_partial_ = -0.93, *t*_122_ = -2.41, *p* = .02) and the Psychotic-like Experiences group (β_partial_ = -0.67, *t*_121_ = -1.82, *p* = .07) gave more positive/less negative ratings for scenes without people. At-risk groups did not differ from each other in their valence ratings of objects, scenes without people, or scenes with people (all *p*s > .49). With respect to arousal, both scenes without people (β_partial_ = 0.70, *t*_122_ = 2.76, *p* = .007) and scenes with people (β_partial_ = 0.82, *t*_122_ = 3.23, *p* = .002) were rated as more arousing than images of objects for the Control group. At-risk groups did not significantly differ from the Control group, or with each other, in their arousal ratings of objects, scenes without people, or scenes with people (all *p*s > .18).

**Appendix**

**IAPS Images Used by Category**

**Neutral Images**

Objects: 7000, 7002, 7004, 7006, 7010, 7025, 7034, 7035, 7040, 7041, 7056, 7090, 7100, 7150, 7175

Scenes without People: 5390, 5471, 5510, 5530, 5531, 5731, 5740, 7490, 7491, 7500, 7546, 7547, 7590, 7595, 7700

Scenes with People: 2102, 2190, 2191, 2200, 2214, 2280, 2305, 2357, 2381, 2383, 2385, 2393, 2512, 2570, 7550

**Pleasant Images**

Exciting: 8030, 8080, 8180, 8185, 8190, 8200, 8210, 8300, 8496, 8370, 8380, 8400, 8470, 8490, 8031

Affiliative: 1441, 1463, 1710, 1750, 1920, 2040, 2070, 2071, 2080, 2091, 2150, 2165, 2340, 2345, 2550

Erotic: 4608, 4650, 4652, 4658, 4659, 4660, 4664, 4670, 4676, 4680, 4687, 4689, 4690, 4694, 4695

**Unpleasant Images**

Disgusting: 2730, 2981, 7380, 9008, 9040, 9140, 9181, 9300, 9301, 9320, 9373, 9561, 9570, 9571, 9830

Threat: 1120, 1300, 1301, 1930, 2811, 3530, 6250, 6312, 6313, 6315, 6370, 6550, 6560, 6571, 9425

Mutilation: 3015, 3016, 3030, 3051, 3101, 3102, 3110, 3120, 3140, 3168, 3170, 3190, 3261, 3266, 3400
